# Toward Sensor Measurement Reliability in Blockchains

**DOI:** 10.3390/s23249659

**Published:** 2023-12-06

**Authors:** Ernesto Gómez-Marín, Luis Parrilla, Jose L. Tejero López, Diego P. Morales, Encarnación Castillo

**Affiliations:** 1 Infineon Technologies AG, 85579 Neubiberg, Germany; ernestogm@correo.ugr.es; 2 Departamento de Electrónica y Tecnología de Computadores, Universidad de Granada, 18071 Granada, Spain; joseluistejero@correo.ugr.es (J.L.T.L.); diegopm@ugr.es (D.P.M.); encas@ugr.es (E.C.)

**Keywords:** Internet of Things (IoT), blockchain, smart contract, hardware oracle, public key infrastructure (PKI), trustworthiness

## Abstract

In this work, a secure architecture to send data from an Internet of Things (IoT) device to a blockchain-based supply chain is presented. As is well known, blockchains can process critical information with high security, but the authenticity and accuracy of the stored and processed information depend primarily on the reliability of the information sources. When this information requires acquisition from uncontrolled environments, as is the normal situation in the real world, it may be, intentionally or unintentionally, erroneous. The entities that provide this external information, called Oracles, are critical to guarantee the quality and veracity of the information generated by them, thus affecting the subsequent blockchain-based applications. In the case of IoT devices, there are no effective single solutions in the literature for achieving a secure implementation of an Oracle that is capable of sending data generated by a sensor to a blockchain. In order to fill this gap, in this paper, we present a holistic solution that enables blockchains to verify a set of security requirements in order to accept information from an IoT Oracle. The proposed solution uses Hardware Security Modules (HSMs) to address the security requirements of integrity and device trustworthiness, as well as a novel Public Key Infrastructure (PKI) based on a blockchain for authenticity, traceability, and data freshness. The solution is then implemented on Ethereum and evaluated regarding the fulfillment of the security requirements and time response. The final design has some flexibility limitations that will be approached in future work.

## 1. Introduction

Currently, the supply chain plays a fundamental role in modern industries [1]. Companies are undertaking significant efforts to update their supply chains with new technologies to increase their competitiveness [2]. In order to establish modern supply chains, the combination of blockchains and the Internet of Things (IoT) is being considered because the integration of these technologies offers an array of benefits that can significantly enhance overall performance [3].

Many studies have been researching their potential and analyzing their impacts. For example, Vicenzo V. et al. illustrated in [4] that using reliable measurements from IoT sensors can detect non-compliant products in supply chains, as well as economically optimize product management by up to 63% (even for cheap and non-critical products). Other investigations show that they can potentially reduce world hunger, [5], or they can be used for legitimately selling users’ information [6]. Also, other publications have been researching their limitations and improvements, like in [7], where it is explained how to analyze the quality of the measured data in a coldchain. Also, the work of Hiu H. et al. [8] explained how the reliable data obtained through blockchains and the IoT can be processed with machine learning to obtain very valuable information. Moreover, the combination of the IoT and blockchains has proven to be extremely advantageous in numerous surveys in the field [9,10,11].

This combination is fruitful due to the synergy of the two technologies in fulfilling the requirements of the supply chain realm. Indeed, a supply chain involves multiple stakeholders with diverse interests, and the products it oversees are subject to information demands from various parties. In these complex scenarios, IoT sensors can provide extensive real-world data. Furthermore, blockchains, which are decentralized and transparent platforms, can corroborate the fact that there is no illegitimate data manipulation on the cloud. They also propose a method for applying transparent and consensual logic operations over data on the cloud, called smart contracts. This symbiotic relationship offers stakeholders traceable insights into the real-time dynamics of products; meanwhile, it also enhances the reliability and accountability of the supply chain [9]. However, these two technologies have important drawbacks that must be addressed. Even if the IoT and blockchains are crucial to ensuring the viability and security of supply chains, their combined implementation is not a trivial task.

Blockchains are a type of complicated infrastructure that require perfect synchronization between multiple nodes. More importantly, the reliability of their data and operations ultimately rely on the trustworthiness of those uploading the data, the Oracles, and the reliability of their data [9]. This is a known blockchain drawback called “the Oracle problem” [12]. Essentially, it implies that blockchain operations and data are as trusted as the Oracles themselves. Many proposals have been trying to address this problem and send reliable information to blockchains like [13,14,15,16], but they were not originally designed for implementation with the IoT. This limitation arises due to the inherent absence of trusted relationships within IoT systems. It is very difficult to ensure the credibility of these devices, the correct device status (trustworthiness), and no tampering with the data flow [17,18] since these devices are inherently insecure [19]. The distrust of the IoT very negatively impacts the motivations for blockchain and IoT adoption because the most predominant reason for adopting blockchains in supply chains is trust [3]. This ultimately affects the potential benefits that could bring the use of these technologies to supply chains.

Despite this, a limited number of studies are planning a solution for securely sending data from IoT devices to blockchains. However, some have not even devised a mechanism for blockchains to identify IoT devices like [20,21,22]. The work of Jonathan et al. [23] do indirectly identify these devices, but it requires a huge setup effort per sensor enrollment. In any case, none of them have proposed a solution for blockchains to corroborate the trustworthiness of the sensor itself when the data were gathered.

In our proposed work, we propose a solution for all these problems. Firstly, we propose the use of secure hardware architecture [24] that signs the data even before being gathered by the IoT nodes, thus achieving the much sought after trustworthiness. Secondly, we combine that secure sensor with a personalized blockchain-based PKI to enroll and identify the IoT nodes quickly and cheaply. And, finally, we devise a new method for ensuring the data are fresh when uploaded to a blockchain. This is groundbreaking solution because it allows blockchains to efficiently validate the reliability of the data generated by IoT devices before accepting it. By doing so, stakeholders in the supply chain can access real-time information about the products with the sought after level of reliability.

The remainder of this paper is structured as follows: Section 2 defines the methodology for analyzing the reliability of incoming data and explains the related state-of-the-art (SoA) work on this topic. Section 3 details the information needed to understand the proposed work. Section 4 presents the details and challenges of the cold chain used as the use case for the proposed solution. Then, Section 5 explains, firstly, how the system works and, secondly, explains the details of the design. Section 6 describes our real implementation in a controlled environment. Section 7 analyzes our proposal using a detailed methodology, as well as compares the results with the SoA works. Section 8 offers a comparison of our work with the SoA works. Finally, Section 9 closes the paper by summarizing the results, contributions, and future work.

## 2. Related Work

For the implementation of trusted hardware, the Oracle requires a comprehensive approach as each step in the data flow must be secured in order to trust the final data [25].

As a first step to analyzing the related work and later comparing them to our proposal, we will establish the security requirements of the information to be processed in Section 2.1 based on the work of Dan Liu [26]. There, we will also discuss how to apply their secure data analytics, which were originally oriented to edge computing, to IoT devices interacting with blockchains. Later, we will use these security requirements to analyze the state-of-the-art works and our development.

In order to analyze the SoA works, we divided this study into three sub-sections.Thus, we firstly explored the research related to the design of a blockchain-based PKI in Section 2.2. Secondly, we delved into studies that use the IoT in blockchains as data providers without security mechanisms (Section 2.3). Lastly, we examined works that designed a secure mechanism to upload general data to blockchains (Section 2.4).

### 2.1. Data Security Requirements

Liu et al. compared, in [26], different works based on edge computing regarding security data. For this comparison, they defined a number of requirements that were divided into three categories: security, privacy, and performance. Despite being a work focused on edge computing, the security requirements were perfectly applicable to analyzing the IoT as an Oracle in blockchains. In total, five security requirements were considered:

- Data Origin Authenticity (DOAu): The authenticity in the infrastructure of a device that generates particular data. This is called authenticity (Au) in [26].
- Data Origin Traceability (DOTa): The capability of the backward identification of a data generator from the data. This is called traceability (Ta) in [26].
- Data Origin Integrity (DOI): The capability of proving that the data generated in a particular point was not manipulated in the course to its final point. This is defined as integrity (I) in [26].
- Data Origin Trustworthiness (DOTu): The capacity to prove that the entity that generated a particular data was not manipulated or attacked, i.e., it was in a trusted status when it generated the data. This is called trustworthiness (Tu) in [26].
- Data Origin Freshness (DOF): The capacity to prove that the data were generated in an absolute timestamp. It is essential to avoid replay attacks and delay attacks (attacks in which a measurement is taken at a particular time, detained, and then published later). This is an additional property that was not included in [26].

### 2.2. PKI in Blockchains

In this section, we will focus on those works that developed specific PKIs for the IoT in existing blockchains. There are many interesting works that have developed an IoT PKI, thereby creating their own blockchain infrastructure or consensus protocol like [17,27,28,29]. However, those works cannot be applied to existing public blockchains such as Ethereum, or to consortium blockchains such as Hyperledger Fabric. In our work, we want to integrate IoT devices to existing blockchains. In this sense, these solutions are out of our scope, and we will focus on those that can be applied to the well-known blockchains.

Stephanos M. and Raphael M. R. proposed IKP [30], a system designed to contribute to the current Transport Layer Security (TLS) PKI. In their architecture, they provide incentives to Certification Authorities (CAs) with correct behavior, while penalizing those with inappropriate practices automatically. While this solution offers a flexible and robust public key infrastructure, it lacks the specific focus on enabling smart contracts to actively interact with the PKI to authenticate IoT devices.

Ankush S. and Elisa B. [31] proposed a system where the hash of the certificate is stored in blockchains together with the ID of the device. In this solution, when an entity needs to check the veracity of a received certificate, they ask a blockchain if the hash of the certificate is reliable or not. The advantage of this system lies in the dynamic revocation and addition of certificates.

Alexander Y. et al. [32] proposed a system to implement the classic certificate chain of trust in Solidity (the programming language of Ethereum’s smart contracts). Each CA has its own smart contract, where it uploads its certificate and stores the hashes of the certificates it issues. On the other hand, it requires modifying the X.509 standard [33] with some minor additions.

All these systems require the use of X.509 certificates to identify devices. These certificates are heavy (Google’s certificate is 1.13 KB, for instance), and are complex to process in Solidity because of the absence of core libraries for string manipulation [32]. The certificates would need to go along with each transaction to identify the device, as well as be verified by the smart contract itself in each transaction, which would increase the cost in each transaction.

To avoid this, we propose a system without certificates using what we define as a Smart Certificate Authority (SCA). The SCA is deployed as a smart contract that checks if an entity meets certain requirements, and, if so, instead of delivering a certificate, it simply stores the address of the entity along with its attributes. Due to the qualities of smart contracts, if an entity has been identified and authenticated by a smart contract, this process is trusted by the rest of the blockchain and does not have to be repeated (verify once, authenticate any-when). As a result, any smart contract that wants to authenticate an entity simply has to query the SCA if the identity is stored, thus avoiding duplicate certificate verification in each communication.

### 2.3. The IoT in Blockchains

In this section, we discuss those papers that use the IoT as a service for blockchains and how they solve the identification problems posed by the IoT.

Little information on this topic can be found in the SoA works, as highlighted by the study conducted by Mohamed Laarabi in [34] on March 2022. In their study, only two articles were detailed with scenarios where smart contracts receive data gathered from sensors [6,21].

In [21], the authors proposed a system for managing the energy consumption of IoT actuators based on the measurements received by the IoT sensor. In this work, they did not propose the identification method of the data in the smart contracts, but they stored the public credentials and signatures along with the data in blockchains, as well as left the actuators as responsible for somehow identifying the data. Thus, they used blockchains as a database. In our work, we developed an infrastructure in which the smart contract itself identifies the senders.

The main contribution of Carlos Molina-Jimenez et al. in [6] was highlighting that conventional business contracts can be automated using centralized applications, decentralized applications, or via combining both. Also, they focused on the complexity of the last one. The work was presented using the example of selling to a customer Alice’s personal information, which was obtained through her IoT sensors. Data security, however, is not covered within the scope of this project.

Another work, proposed by Mohamed Ahmed et al. [7], focused on finding, defining, and proposing systems for measuring the quality dimensions relevant for IoT data qualification. This work presents the context of a medical equipment cold chain, where IoT nodes provide the smart contracts with qualification data. It is the same use case where we will present our work, as detailed in Section 4. In this use case, Mohamed Ahmed et al. defined four main data quality dimensions: accuracy, completeness, consistency, and currentness. Also, they proposed a method to calculate them. But, as they recognize, the IoT data sources’ security was a field that was not embarked upon in their work and yet must be addressed. This is where our research comes in, i.e., ensuring the non-manipulation of devices or their messages. However, it is outside of our scope to evaluate the quality dimensions of the messages. As such, we consider that work as having a great synergy with our proposal.

The work from Zheng Zhang et al. [35] presented a framework for trustless data sharing based on blockchains to reduce the risk of data tampering. It combined the layers of IoT, fog computing, micro-services, and decentralized applications to offer services to smart contracts on blockchains. They act as a platform as a service, and work as application programming interfaces (API) for smart contracts. It is an interesting improvement for blockchain applications. However, as they declare, the more crucial problem in their solution is the lack of IoT security.

A more recent article from Faheem A. R. et al. [36] proposed a secure and manageable mechanism through which to share electronic health records. The solutions used blockchains to protect the integrity of the electronic health record, including te health data gathered by IoT devices. The work focused on developing an interoperable framework to reliably share health records between systems and providers with patient authorization. However, that work did not pay any attention to the security of the IoT devices or their data.

### 2.4. Oracles

In this subsection, we will examine the research conducted on uploading trustworthy information to blockchains. The purpose of this effort is to enable smart contracts to depend on this data, thus ensuring stakeholders can confidently execute high-impact tasks.

There are several Oracles designed to upload specific information that are excluded in the analysis since they cannot be directly applied to the IoT, like PriceGeth [15] used to publish price pairs or Augur [16] for market prediction.

Some of the SoA works found in that field propose servers or clients to feed smart contracts, never directly from IoT nodes, i.e., the smart contract never verifies the signature performed by the IoT device (edge-to-edge signature). Moreover, just one of them considered the integrity of IoT nodes.

Zhang et al. [13] proposed a system to feed smart contracts with information from reliable web pages using HyperText Transfer Protocol Secure, which was achieved by assuming that if these web pages are reliable for non-blockchain applications with high impact, then blockchain applications can also use them. The system is called Town Crier. In this system, Intel SGX is used to guarantee the correct operation of the Oracle, which is not responsible for the reliability of the data but the correct data source. The correctness of the data is guaranteed under the assumption of the validity of the data source, i.e., reliable web pages. In their paper, DOAu, DOTa, and DOI are guaranteed through the use of off-chain TLS certificates and Intel SGX-based remote attestation. In the case of DOTu, it was achieved through the confidence that resides in the websites, and, finally, the DOF is provided using SGX clocks and a public timestamp verification. This infrastructure is complete, but it can not be applied to the IoT since DOAu and DOTu are achieved by the general knowledge and trust in the data generators, i.e., the websites, which cannot be applied to the IoT. The same problem was found in Chainlink [37].

DiOr-SGX [20] has similarities to Town Crier [13] because it uses Intel SGX to ensure the correct functioning of the Oracle, but it differs by creating a decentralized system to ensure availability, as well as adds a voting system and prestige rewards to choose the leader of the Oracles with the best response time. In this system, the smart contract generates an event to request data. Then, this event is read by the Oracle leader, who requests the data from other Oracles (Oracle nodes). These Oracles collect data from IoT nodes and send it to the leader along with proof of their correct operation through Intel SGX. This system is focused on promoting the best self-organization for acquiring the shortest response time. Also, the leader performs a remote attestation process on the other Oracles to make sure that they did not manipulate the data. But nothing verifies the leader, and it does not provide any mechanism through which to ensure the veracity of the data, i.e., it does not offer any mechanism for DOAu, DOTa, DOI, DOTu, or DOF because, although there is a mechanism to check the correct status of the Oracles nodes, none of them can identify the origin of the data received by the Oracle nodes. On the other hand, there was no penalty mechanism found for those who deliver data different from the average. Finally, this system, due to its decentralized nature of distributed data collection, where many nodes shall obtain the same data from different sources, can be applied in use cases such as the temperature of a city, but it can hardly be applied to a cold chain where all the nodes that measure the temperature belong to the same entity.

Astraea [14] is a mechanism for contributing binary information (true or false) to blockchains. The information is provided through a system of voting and certification. All “players” have to contribute an amount of money to vote or certify, and they lose money or are rewarded according to the data provided, which motivates them to behave honestly. It is impossible to know where the data ultimately come from in this solution; therefore, it there are no DOAu and DOTa. The DOIO, DOTu, and DOF are guaranteed through economic rewards and penalties. Also, the solution can easily enroll new players, which makes the solution scalable. The problem with this system is that it is only applicable to decidable and verifiable information that is accessible to a high number of players from different sources. However, this condition is not applicable in all scenarios. The same problem was found on other Oracles based on reputation or a voting system [38].

In Jonathan Heiss’ work [23], they proposed two different systems to gather signed data from a sensor through a gateway that processes and sends it to blockchains. The smart contract itself can check the correct processing of the incoming data using ZoKrates in the first solution, and the Intel SGX-based remote attestation in the second solution. In both of them, the gateways process the IoT data, which is achieved by considering the verification of the IoT signature as part of the data processing. Then, the smart contract attests the correctness of the data processing, and, because the IoT signature verification is included in data processing, the smart contract then indirectly verifies the IoT signature. This system provides DOAu, DOTa, and DOI. However, both mechanics require a trusted and critical setup that is not explained in their proposal. They assume the existence of a trusted setup in every enrollment that can be verified by each stakeholder, thus making its real implementation very complex. Moreover, there was no process outlined to probe the non-manipulation of the IoT node (DOTu). Finally, with no further details, the gateway accepts any signed data from the sensor so that old signed data can be accepted (DOF).

The work of Alia Al Sadawi et al. [22] is the only SoA work that claims being the first study that alone detailed a entire process for the integration of the IoT in blockchains. This was achieved through the use of a hardware Oracle with cryptographically attestable and anti-tampering properties. This secure IoT device measures CO_2_ levels and signs the outgoing data with a nonce. The information is sent to a fetching script that writes it on a blockchain through a transaction. At the end of the document, the authors performed a detailed security and vulnerability analysis to ensure the robustness of the smart contracts but not of the full system. Additionally, there were no details for a public attestation procedure of the hardware Oracle, so there was no mechanism for proving to the infrastructure the trustworthiness of the attestable IoT device (DOTu). On the other hand, they did not provide details of any PKI or a similar system to authenticate the IoT nodes; therefore, there was no DOAu. Additionally, the measured data passed through a fetching script (e.g., a Python script), which sends it to a blockchain, and the owner or an attacker could manipulate it to send any arbitrary data, thus losing DOI. Finally, even when using a nonce to avoid digital signature repetition, the data could have been gathered and signed at any moment, thus they were vulnerable to delay attacks (DOF).

Even though hardware Oracle is a known category [39,40] with its own standard and qualification analysis [7,25], and which is included in surveys such as [41,42], to the best of our knowledge, there is just one SoA implementation that lacks some important details like the PKI and does not meet several security requirements. To the best of our knowledge, our paper presents the only infrastructure capable of providing IoT-generated data directly to the smart contract with an edge-to-edge signature, where a blockchain can verify DOAu, DOTa, DOI, DOTu, and DOF with a dynamic enrollment process, and is applicable to Ethereum.

## 3. Background

In this section, we will provide some background for a better understanding of the proposal developed in this work. Thus, we will introduce the concept of a Secure Sensor, some details regarding Ethereum, and how smart contracts are implemented through a blockchain.

### 3.1. Secure Sensor

Dominic Pirker et al. [24] presented four novel solutions for achieving unquestionable trust in the measurements obtained by an IoT device. We will consider their solution “Concept A” for our work, and, in the following, it will be referred to as Secure Sensor (SS). In an SS, we have to differentiate three different elements:

- Controller: the core of the IoT node itself that, through a Turing machine, can perform any task.
- Sensor: the hardware extension connected physically to the controller that—through SPI, I2C, or buses—receives commands and sends the measured data.
- Hardware Security Module (HSM): a hardware module secured by design with the capacity to create private–public key pairs, as well to store and use them.

**Figure 1:**
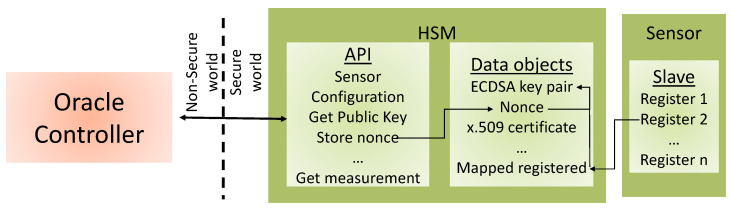
High-level schematic of a Secure Sensor. The controller can only interact with the HSM through a limited API. The measurements gathered by the sensor and a nonce are signed with a sealed key before they are sent to the controller.

Thus, a SS is an IoT device with a controller, a sensor, and a HSM. The distinctiveness of a SS from other IoT device architectures is the fact that the controller cannot communicate directly with the sensor but the communication is done through the HSM. As is shown in Figure 1, the Oracle controller can interact with the HSM through a limited API. The HSM is in charge of gathering the data from the sensor, signing that data using a nonce, and forwarding it to the controller together with the digital signature. The private key used for this digital signature is a sealed key, which means that it cannot be used for any other purpose. Because the element that generates the data is hardware-protected (shown green in Figure 1), this device provides DOTu.

The downside of this work is the complexity of distributing the public keys required for DOAu, as well as in implementing a verifiable random nonce for introducing the DOF into measurements.

### 3.2. Ethereum Addresses

Ethereum is the most popular blockchain for IoT applications, as well as for smart contracts in general [9]. Ethereum uses the Elliptic Curve Digital Signature Algorithm (ECDSA) with a 256-bit-long private key and, consequently, a 512-bit public key. The Ethereum address associated with this private key is composed of the last 160 bits of the Keccak [43] hash of the public key. Therefore, a key pair has the following elements: a private key (Priv), a public key (Pub), and an address. In this way, from a signature or a public key, the address can be easily derived, and we do not have to store 512-bit long public keys. Thus, in our approach, which was developed over the Ethereum platform, we used the Ethereum addresses as identifiers of the entities. Following Hilarie Orman’s words [44]: “Who am I? you are your Blockchain address”.

### 3.3. Blockchains and Smart Contracts

As was commented in Section I, blockchains are a system developed by Shatosi Nakamoto at Bitcoin in 2008. They serve as a distributed consensual peer-to-peer database [45], a perfect environment that includes “smart contracts”. It is a concept that was defined by Nick Szabo in 1997 [46] to formalize and secure relationships over computer networks. But, it was not until 2014 that smart contracts were implemented in Ethereum [47], thereby allowing one to execute scripts in a public blockchain that is similar to Bitcoin and satisfies the definition of Nick Szabo, thus implementing real smart contracts.

Data in Ethereum are organized in blocks. The blocks are identified by the block’s number or the block hash. The last is generated through the hash of all the data in the block. The nodes add new blocks to update the database without deleting previous ones. Every block is known as the “father” of the next one. The time interval between the blocks’ generation is called block time. The consensus protocol defines the entity that adds new blocks to the chain. Bitcoin’s consensus protocol is Proof of Work (PoW) [48], just like what Ethereum’s was initially. In PoW, each new block proposes a mathematical problem that takes an average time equal to the block time to be solved. The first node to solve the problem (the miner) publishes the new block, where it includes the solution and the time at which the generation of the block started (the block timestamp). In PoW, the miner has some freedom in setting the block timestamp, which makes the block timestamp unreliable [49,50].

In September 2022, Ethereum migrated through a complex process known as “The Merge” [51] to a different consensus protocol named Proof of Stake (PoS) [52]. To participate in this consensus protocol, the interested entities have to stake Ethers, the crypto-coin used in Ethereum. In this way, only these entities, named validators, can propose and validate blocks. For a particular block, these validators are randomly selected by “The Beacon Chain” [53]. In this new consensus protocol, blocks can be added every 12 s, and the block timestamp is strictly defined by the slot in which the block is published, thus avoiding any subsequent alteration by the validator. However, the slots may not contain blocks if the selected proposer does not propose one.

**Figure 2:**
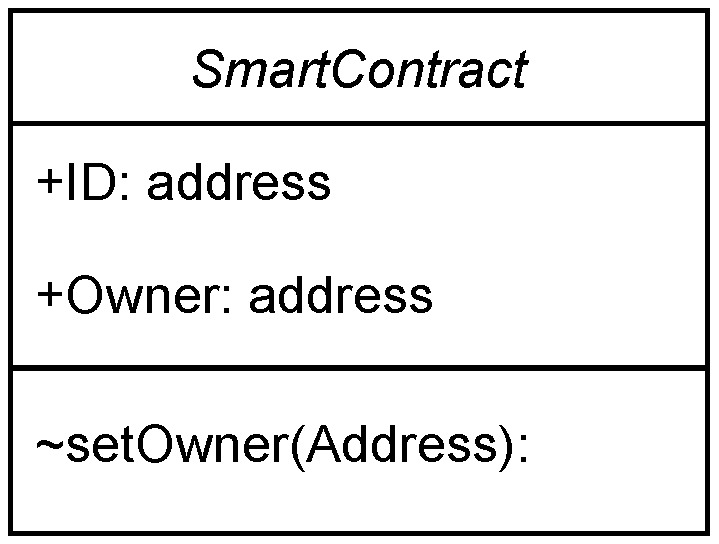
Abstract class “Smart contract” following the Unified Modeling Language.

Smart contracts executed on PoS provide the confidence of knowing that 2/3 of the network has validated its execution. However, the features provided by smart contracts under PoS have not been updated from previous versions of Ethereum, which were based on PoW to ensure backward compatibility. Smart contracts use addresses as identifiers, like in the case of the users. In order to ease the management of smart contracts, it is normal to define a user with special privileges, thus allowing them to modify some of the settings and data involved in them. This user is called the owner. Figure 2 shows the abstraction diagram of our smart contracts following the Unified Modeling Language (ULM) where the ∼ symbol denotes that the method is only accessible to the owner.

The operation with smarts contracts is based on triggering logical operations through signed transactions [54]. The execution of these logical operations implies a computational cost that, in Ethereum, is measured in specific units named *gas* [55]. Due to consensus and verification mechanisms, smart contracts are executed in a huge number of nodes simultaneously; thus, the gas required by them can be very expensive. As a consequence, optimization of the computation costs of smart contracts is a priority. Multiple transactions are grouped in a block that is then stored on a blockchain. Regarding the modifications on the ledger, which are called transactions, they are grouped into blocks, which are later stored in a blockchain. All transactions follow the same structure, and the more relevant fields when working with smart contracts include the following:

- Raw transactions:
  – Sender: address of the transaction’s signer.
  – Addressee: address of the transaction recipient.
  – Data: name of the calling functions and variables.
- Signature: signature of the raw transaction.

### 3.4. Assumptions

In order to use Secure Sensors as the source of trusted data, it is required to make some assumptions, which are the following:

- Trusted manufacturer: The manufacturer of SSs is well known and public. In this way, it can certify the correct manufacturing of the device. It is a common assumption in HSMs, e.g., the endorsement certificate in Trusted Platform Module 2.0 [56], which is a standard for crypto processors.
- Trusted smart contract: The smart contracts, being part of our solution, shall be free of bugs and verified by all the stakeholders before and after they are deployed in a blockchain.
- No undetectable attacks to SSs: The HSM included in a SS is secure by design, thus it will avoid any software attack. Additionally, physical attacks will trigger hardware protection mechanisms, thus leading the device to become useless.

It is unnecessary to assume an invulnerable or reliable microcontroller in a SS. Our solution will not be at risk even if an attacker can control it fully.

## 4. Use Case: Ensuring the Respect of the Cold Chain through Smart Contracts

As a use case for presenting our proposal, we will use the scenario of a cold chain, where the transported goods must maintain strict temperature conditions. In this scenario, also used by Ahmed et al. [7], the correct fulfillment of these conditions is essential for the product value. Furthermore, in the cited work, due to its proximity to a real business, the authors were provided with the actual strict temperature conditions of a medical product for blood testing. The non-accomplishment of this compliance requirement could lead to a breakage of the product. Therefore, in this scenario, not only the product distributor, but also the complete supply chain is responsible for the quality of the product.

There are at least four stakeholders in our scenario: the shipper (the originator of the transport request), the carrier, the receiver, and the IoT manufacturer (in charge of manufacturing the temperature sensors). The transported goods have temperature sensors with internet access (IoT nodes).

In this context, the process is started by the receiver, who requests a product with quality requirements. Then, the shipper accepts the request by offering a product that meets the rates if it stays within the threshold temperature during transport. Next, the carrier accepts the thresholds, and, finally, the three agree on the penalties for infringement and choose a manufacturer for the IoT nodes.

However, if there is not enough confidence in the reliability of the system, there will be no interest in the infrastructure. The following are the risks we identified in the use case:

- Sensor replacements with other IoT devices that could generate invalid data (DOAu).
- The origin of all valid data has to be identified by the unique data generator (DOTa).
- Manipulation of the data collected by the sensors (DOI).
- Software manipulations of the sensors (DOTu).
- Time modifications with which the IoT nodes collected the measurements (DOF).

In summary, such a use case essentially requires the DOAu, DOTa, DOI, DOTu, and DOF of the IoT nodes so that the stakeholder can trust the system and they can set a smart contract for the enforceable agreement.

## 5. Design of the Proposed System

We firstly explain a high-level view of our solution in Section 5.1. Secondly, the details of the PKI required for the system are explained in Section 5.2; next, the proposed solution to guarantee the freshness of the measurements is detailed in Section 5.3. Section 5.4 shows how to insert reliable information in the blockchain, and, finally, the complete process is presented in Section 5.5.

### 5.1. Proposed System

**Figure 3:**
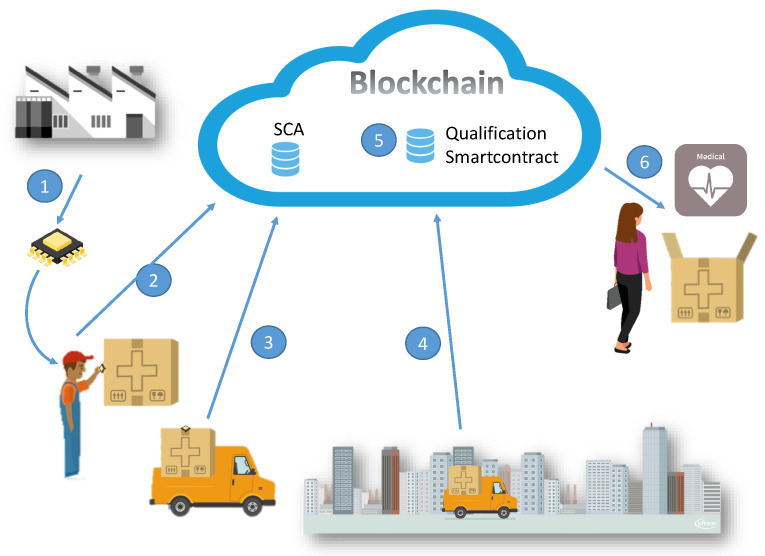
High-level scheme of a SS installed in a package of a supply chain sending data to a blockchain.

In our secure system for achieving reliable measurements from IoT nodes, the process starts with a setup phase where the stakeholders agree on the conditions of the smart contracts. In the use case that was used as an example, this setup phase will imply the agreement on the cold-chain conditions by generating the qualification smart contract. In this smart contract, the stakeholders stipulate who will be the manufacturer of the sensors, the sensor model to be used, and other legal information about the sensor (recalibration, digital certificate, or digital certificate by accreditation institution [25]). Then, they generate the SCA smart contract. The proposed framework is depicted in Figure 3. This figure shows the lifecycle of a SS that was used to track the temperatures of a medical supply, starting at the manufacturer and finishing with the final customer. Each of the steps, one through six, is explained in more detail below.

1. First, the sensor manufacturer generates, signs, and delivers a certificate to each device manufactured. This is the manufacturer certificate.
2. The shipper receives the sensor and prepares the package with the device, which will have to maintain a specific temperature throughout the entire cold chain. Then, the shipper pre-registers the package on the blockchain with the package ID and the SS address.
3. The sensor then asks to be publicly identified and published in the SCA, thus creating a transaction that includes the manufacturer certificate. The SCA then initiates the verification process with a moderate gas cost. It will check that the identification request and the manufacturer certificate meet all the requirements, and, if the request is valid, the SCA will then store the SS address as a trusted address along with important information data about the sensor. Therefore, if an address is stored in the smart contract, it means it has passed successfully through the verification. This completes the registration in the PKI needed for DOAu.
4. When the SS uploads a data package to a blockchain, it will first read a recently published nonce, which is explained in more detail in Section 5.3. Then, the SS will sign the measured data together with the nonce. Data and signatures are added inside the transaction, and then signed again and sent to a smart contract called a Qualification Smart Contract (QualificationSC).
5. Upon receipt of the transaction, the QualificationSC will check that the KeyPair that signed the transaction has its address stored in the SCA (thus obtaining the DOAu). If yes, it will verify other elements, such as the following: the SS that was used to sign the data (DOI and DOTu), and whether the nonce included in the data signed was fresh (DOF). There is no need to verify any certificate in this step.
6. Finally, the receiver, when receiving the package, reads the address of the sensor in the package and looks for the package’s qualification data in the blockchain.

### 5.2. Public Key Infrastructure Used

The goal of our system is to make smart contracts that are capable of checking the origin of received data. In our scenario, these data come from a SS, as described above.

As detailed in Section 3.1, the SS had a particular architecture, as shown in Figure 1, where three modules were specified: the Oracle controller, HSM, and sensor. The device generates a public–private key pair (KeyPair) with special features, the secure element KeyPair (SeKP). The private key (Priv) is always stored in the HSM, and it can only be used to sign the data coming from the sensor and the nonce delivered from the microcontroller. The DOTu and DOI of the signed data can be verified by verifying a signature that is generated using this key.

**Figure 4:**
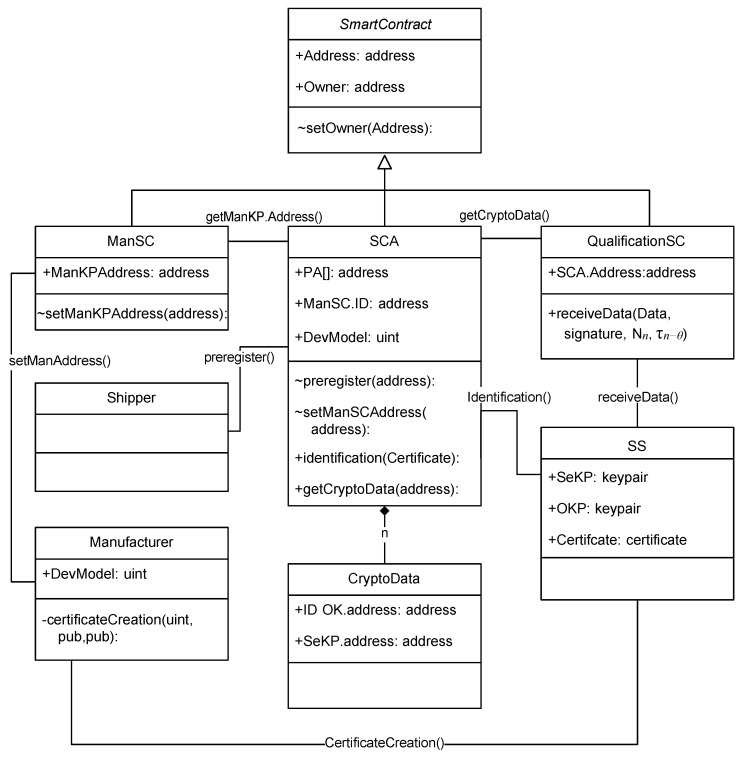
Class diagram of the complete infrastructure following the UML.

The device will interact with a blockchain by sending and signing its transactions. The transaction structure is generated in the Oracle controller, which performs the transaction hash and sends it to the HSM that signs it. However, due to the previously explained security limitations, SeKP cannot be used to sign hashes that are generated externally.For this reason, a second KeyPair is needed to sign them. This second KeyPair without limitations is called the owned KeyPair (OKP), and it is used exclusively to sign the hashes of the transactions for a blockchain. Finally, these two KeyPairs are linked to each other and to the manufacturer through a certificate called the manufacturer certificate. The three data components, SeKP, OKP, and the certificate, are stored by the device SS, as can be observed in Figure 4.

The manufacturer certificate contains the device model (ModelDevice), the SeKP.Pub and OKP.Pub keys, and its signature. The manufacturer will keep the address of its signing key (ManKP.Address) updated in a smart contract, i.e., the manufacturer smart contract (ManSC), as can be observed in Figure 4. Note that the ManKP.Address can be updated only by the manufacturer (who is the owner of the ManSC).

The final part of our blockchain-based PKI is the Smart Certificate Authority (SCA). In this smart contract, the shipper can preregister its devices. Later, the SS can request an identification from the SCA. The last one will check the requestor’s information upon receiving a transaction. Then, the SCA will transparently verify the manufacturer’s certificates and other information about the sensor. If, and only if, the requestor satisfies all the requirements will the SCA automatically store a copy in the smart contract of the validated and trusted cryptodata: the OKP.Address and the SeKP.Address. Later, any smart contract like the QualificationSC can consult the SCA for the cryptodata to assert if a SS passed through identification or not. All these actions and the data are graphically detailed in Figure 4. This mechanism has several advantages versus the classic certificate system:

- Due to the features provided by smart contracts, they are as reliable as a certificate signed and validated by all of the blockchain infrastructure that follow the SCA’s stipulated rules.
- Any entity with blockchain access can verify an identity, including the smart contracts themselves.
- Because of blockchain decentralization, this method has a very high availability.
- There is no need to keep an updated revocation list because the address stored in the smart contract can be dynamically removed.
- There is no need to verify a certificate because the response of a SCA is always trusted. It reduces the computer processing consumption, which is essential in smart contracts.

The SCA receiving a certification request will check the following:

1. The manufacturer certificate was signed by the manufacturer.
2. The OKP.pub was preregistered by the shipper.
3. The model device (IDmodel) of the Secure Sensor was the one selected in the setup phase.

### 5.3. Freshness

This subsection will detail the method designed to guarantee the freshness of the actual data. As explained in Section 3.1, an SS includes a nonce in the signature when it gathers data. To guarantee the data freshness, the nonce must be unknown until it becomes publicly known at a time $\tau_{l}$. When the actual data *i* is made public at a time $\tau_{r}$, including the nonce data signed, it is guaranteed that the data were generated in the uncertainty interval $\Delta \tau_{i}$:

(1) $$\Delta \tau_{i}=\tau_{r}-\tau_{l}$$

In our infrastructure, we use the block hash as a nonce. In the Ethereum PoS, the blocks can be published in any slot. A new slot is available every $\Delta \tau_{b}=12$ s, which is called block time. On the other hand, the block hash is generated from the hash of the data, which isincluded in each block. It is important to note that one of the elements that form the block data is a random variable named RANDAO mix ($Rm_{n}$). $Rm_{n}$ is included in the computation of the block hash, and it replaces the variable *mixHash*, which is deprecated after the merge [57]. Notice that using PoS as a consensus protocol implies that the block hash of the blocks cannot be considered random anymore because the proposer can generate several blocks internally and publish the one that interests them the most. This means that smart contracts cannot use the block hash for use cases such as lotteries; instead, they have to directly use $Rm_{n}$. However, even if the block hash is not a random number anymore, it is still an unknown number until the moment the $Rm_{n}$, which belongs to the previous block, is published. For this reason, the block hash can be used as a public nonce for the SS. Nevertheless, analyzing the predictivity of $Rm_{n}$ is important before using a block hash as a nonce. In the following, we will present an analysis of the variable $Rm_{n}$ because its reveal time has the same uncertainty interval as the block hash.

**Figure 5:**
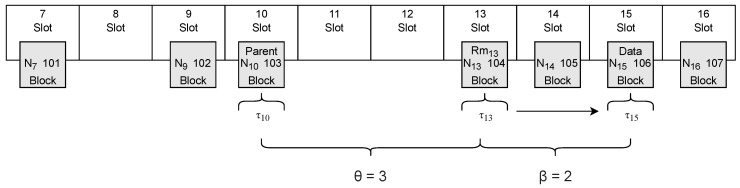
Graphic representation of the variables in Equation (2).

We considered that $Rm_{n}$, when published at block number $N_{n}$ at the slot *n* with a timestamp $\tau_{n}$, can be publicly computed in the moment its parent block is published. Normally, the parent block $N_{n}-1$ is published in the previous slot at slot n−1, i.e., at $\tau_{n-1}$. However, as explained in Section 3.3, slots can be empty if the proposer does not propose any block on time. As such, we defined θ as the difference of the slots between the slot containing the block $N_{n}$ and the slot containing its parent block $N_{n}-1$. That means that $Rm_{n}$ is revealed at time $\tau_{n-\theta }$ (i.e., $R\left(Rm_{n}\right)=\tau_{n-\theta }$). Therefore, using the block hash of block $N_{n}$ at slot *n* as a nonce in a SS when gathering data would mean that $\tau_{l}=\tau_{n-\theta }$. Inserting it in a block at slot n+β, where β∈N>0, would leave $\tau_{r}=\tau_{n+\beta }$, thus obtaining a uncertainty interval that was defined in Equation (2). Figure 5 shows a practice example of this equation, where a SS uses the block hash of block 104 as a nonce to sign gathered data. Then, the data signed are inserted in block 106. The parent of block 104, which is block 103, is inserted into slot 10.

(2) $$\Delta \tau_{i}=\tau_{n+\beta }-R\left(Rm_{n}\right)=\tau_{n+\beta }-\tau_{n-\theta }=\left(\theta +\beta \right)\Delta \tau_{b}$$

However, this uncertainty interval is insecure because $Rm_{n}$ is known in advance by the block proposer of slot *n*. Coordination between the carrier and the proposer can lead to a timestamp attack, thereby allowing for the use of a measurement that was gathered at a time $\Delta \tau_{A}$ before revealing $Rm_{n}$, which is what we call the PrevTime Attack (PTA). The proposer can be elected for several blocks in a row, thus increasing $\Delta \tau_{A}$. Also, there can be accidental empty slots that would help to predict $Rm_{n}$ with a probability of ξ. The probability of being a proposer depends on the amount of money staked in the infrastructure. As μ is the probability of the attacker to be chosen as the proposer of the next block, the probability of knowing $Rm_{n}$ with a time $\tau_{A}$ in advance is equal to the following:

(3) $$Pr\left(\Delta \tau_{A}\right)={\left(\mu +\xi \right)}^{\left(\left\lceil \frac{\Delta \tau_{A}}{\Delta \tau_{b}}\right\rceil \right)}$$

For an attacker investing USD six billion, 16.3% of all the Ether staked, and a 2.9% of the total ether supply at 22 July 2023 [58], μ=0.163 [52]. Also, between 22 July 2023 and 15 April 2023, 1.3% of the slots were empty slots, where ξ=0.013. With this values, a Pr(48)=0.0009 was obtained. With sufficiently low probability, $\Delta \tau_{A}$ can be infinite. Assuming that the carrier always succeeds in performing PTA by obtaining an assumable time $\Delta \tau_{AA}$ such that $\exists \gamma \in N:\Delta \tau_{AA}=\gamma \cdot \Delta \tau_{b}$, then the new minimum uncertainty interval is as follows:

(4) $$\Delta \tau_{i}=\tau_{n+\beta }-\left(R\left(Rm_{n}\right)-\Delta \tau_{AA}\right)=\left(\theta +\beta +\gamma \right)\Delta \tau_{b}$$

where $\tau_{l}=\tau_{n-1}-\Delta \tau_{AA}$ and $\tau_{r}=\tau_{n+\beta }$. Secondly, we assume that the carrier always tries to avoid sending a faulty measurement by using a measurement that takes $\Delta \tau_{NA}$ time longer than $\Delta \tau_{AA}$, such that $\Delta \tau_{NA}\in R>0,\Delta \tau_{A}=\Delta \tau_{NA}+\Delta \tau_{AA}$:

(5) $$Pr\left(\Delta \tau_{NA}\right)={\left(\mu +\xi \right)}^{\left(\left\lceil \frac{\Delta \tau_{A}}{\Delta \tau_{b}}\right\rceil \right)}={\left(\mu +\xi \right)}^{\left(\left\lceil \frac{\Delta \tau_{NA}}{\Delta \tau_{b}}\right\rceil +\gamma \right)}$$

Thus, with μ=0.163, ξ=0.013, and γ=4 (48 s of $\Delta \tau_{AA}$), in order to perform the simpler 12 s attack of $\Delta \tau_{NA}$, we require Pr(12)=0.0002. Although low, this probability is still too high to ignore, but it is easily indemnifiable. To compensate for the probability of 0.02% in terms of performing a successful PTA, each time the carrier inserts an incorrect measurement in the smart contract, it is considered to have attempted an unsuccessful PTA. Then, it shall pay an additional penalty for those times it was successful, equivalent to 0.02% of the package price.

Finally, with this mechanism, QualificationSC can estimate a highly reliable uncertainty interval of the timestamp of the measurements. Each time a SS sends measurements using a block hash as a nonce, i.e., belonging to $N_{n}$, the smart contract will obtain the timestamp of the block $N_{n}-1$ ($\tau_{n-\theta }$); in addition, subtracting 48 s from estimating that the measurement was generated in some moment between the calculated time and the current time, obtains a probability of 99.98%. Still, this solution has a drawback for smart contracts in Ethereum; thus, in the majority of blockchains, it cannot access the timestamp of previous blocks. As such, it cannot access $\tau_{n-\theta }$. To solve this problem, and to avoid using Oracles to provide this data, we developed a novel optimistic approach that is explained in the next section.

### 5.4. Inserting Reliable Information to the Previous Blocks to a Smart Contract

During the execution, a smart contract can access the current time, which in PoS is accurate. Additionally, a smart contract can access the block hash of the last 256 blocks, but it cannot collect any additional data about these blocks like the timestamp. The timestamps of previous blocks cannot be derived using the block numbers because even if the blocks are published in 12 s slots as some slots can be empty without a proposed block. Thus, the time gap between consecutive blocks can be higher than 12 s. An attacker investing USD six billion could easily exploit it to sign at measure at block $N_{n}$ and send it at block $N_{n}+k$ with a real-time gap major than $k*\Delta \tau_{b}$.

Nevertheless, a smart contract can recreate the block hash of block $N_{n}$ in an execution if all the needed data are provided. All the variables that make up a block hash are as follows: ParentHash, UncleHash, Coinbase, Root, TxHash, ReceiptHash, Bloom, Difficulty, Number, GasLimit, GasUsed, Time, Extra, MixDigest, and Nonce. Through using all of these variables and comparing the resulting hash with the block hash $N_{n}$ collected inside the execution, a smart contract can rely on the provided data, i.e., what is in the timestamp. The problem with this method is that the verification process requires high gas consumption (221,570 gas). To reduce gas taxes, we have applied an optimistic approach similar to the one used in the optimistic roll ups [59]. In this approach, all the functions necessary to verify the results of a call are integrated in the smart contract, but this verification is not executed as a general rule to reduce costs. When a function of a smart contract is called externally, the caller directly provides the result, and it is considered valid without going through further on-chain verifications. Then, a time is given for anyone to verify the result off-chain verifications and to denounce the invalidity of the provided value. If this occurs, the smart contract itself verifies the result, reverses the transaction if necessary, and performs the stipulated penalties.

By implementing this approach in our smart contract QualificationSC, the SS itself can provide a timestamp of the parent of block $N_{n}$, in which block hash is used as a nonce in the signing process, where *n* is the slot from where the block hash was gathered. Its parent block was published at slot n−θ. The smart contract relies at first in this value when using it to calculate $\Delta \tau_{i}$. Then, 3 min (15 slots) is provided for any claimer to claim the invalidity of the timestamp provided by the SS and to propose a new one. If someone does this, the person in charge of the sensor (SensorResponsible) can accept the new timestamp, thus avoiding the necessity of reconstructing the block hash and obtaining a significantly reduced penalty. If the SensorResponsible refuses the new timestamp, the claimer can process a “judgment” that provides all the needed information to the smart contract so it can recreate the block hash of $N_{n-\theta }$. If the smart contract can successfully recreate the block hash, meaning that the new timestamp proposed by the claimer was correct, the SensorResponsible has to pay all the expenses transactions and a small penalty. The judgment is a very unlikely call because the SensorResponsible will accept the new timestamp proposed by the claimer if it is correct without the need to go through the judgment process. The judgment costs 240,510 gas, which is equivalent to less than $10 as of 24 July 2023.

With this solution, the SS itself can send the timestamp of the parent’s block, in which block hash is used as a nonce to QualificationSC. Then, the smart contract can trust it without increasing the on-chain costs.

### 5.5. Detailed Process

**Figure 6:**
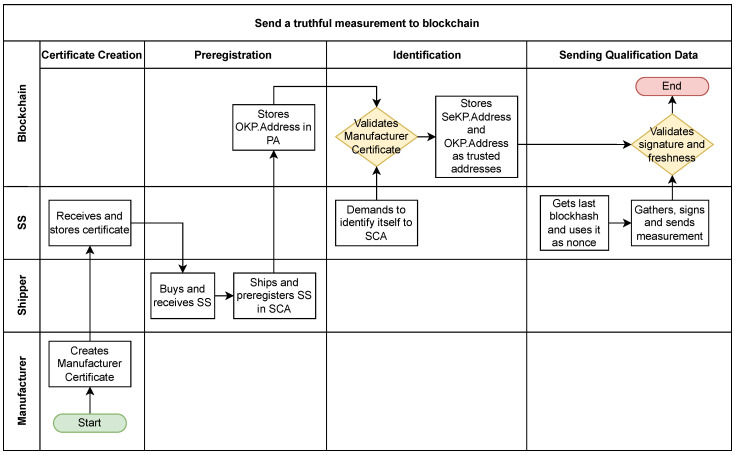
Flow chart of the proposed solution.

In this subsection, we will include a detailed explanation of the PKI in a cold-chain scenario. The flow chart on Figure 6 represents the actors, the phases, and the actions, which are explained step-by-step below.

**Figure 7:**
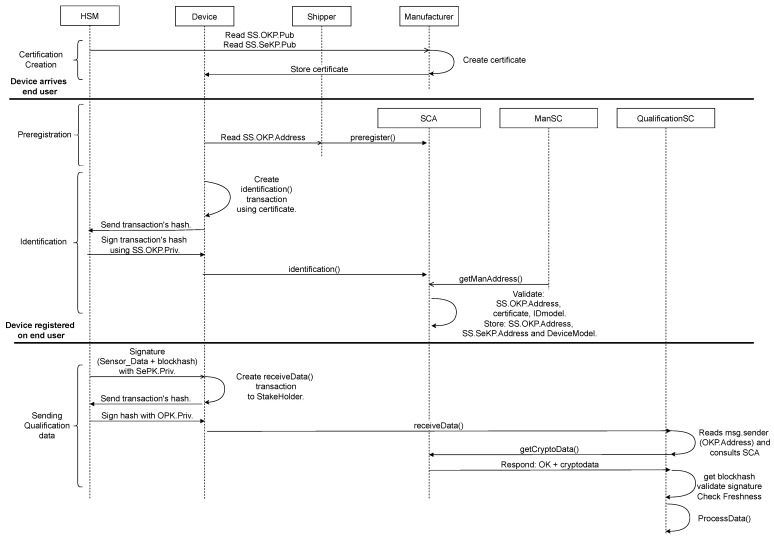
Sequence diagram of the complete infrastructure following the UML.

In the setup phase, the stakeholders must detail the characteristics of the cold chain. They stipulate the manufacturer, the SS model (DevModel), the sensor certificates [25], the assumable time $\Delta \tau_{AA}$, and the qualification requirements. With these data, they can deploy the smart contracts SCA and QualificationSC. Next, the manufacturer deploys its own smart contract, the manufacturer smart contract (ManSC), where it dynamically updates its key used (ManKP) to sign the manufacturer certificates (Cert). Any entity (including smart contracts) can consult the manufacturer’s address in ManSC. After the setup phase, the process sequence starts. Notice that the complete sequence is graphically described in the sequence diagram shown in Figure 7, which details the smart contracts and the actors together with their functions and relationships.

#### 5.5.1. Certificate Creation (*Manufacturer → SS*)

The first phase of the sequence is certification creation in which, once an SS is manufactured, the manufacturer reads its public keys, SeKP.pub and OKP.pub, and creates a certificate including DevModel. Next, the raw certificate is signed using the manufacturer’s private key, MK.priv. Finally, the signature is attached to the raw certificate, thus creating the certificate. It is the only phase that must be performed in a controlled environment, and it is detailed in Algorithm 1.
**Algorithm 1** Certificate CreationInternalInputsMaK_privExternalInputsOKP.pub,SeKP.pub,DevModel      $Cert_{raw}\leftarrow \left(DevModel||OKP.pub||SeKP.pub\right)$      $Signat\leftarrow sign(MaK\text{\_}priv,Cert_{raw})$      $Cert\leftarrow (Cert_{raw}||Signat)$**return** Cert

#### 5.5.2. Preregistration (*Shipper → SCA*)

The SS device is sent to the shipper, who installs it in the package to be shipped and prepares it to start the cold chain. Additionally, the shipper preregisters the OKP.Address in SCA, thereby adding it to a sheet of Preregistered Addresses (PA).

#### 5.5.3. Identification (*SS → SCA*)

Then, the SS request is identified in SCA. A SS uses its OKP.priv to sign a blockchain transaction, which contains its manufacturer certificate. When the SCA receives it, the smart contract first checks that the transaction sender address (Tx.sender address) is in the PA. Secondly, it validates that the manufacturer certificate was signed with the Priv of the manufacturer, whose address is indicated in ManSC. Next, it asserts that the model defined in the certificate, DevMode, is the same model defined in SCA. Then, the SCA generates the OKP.Address from the OKP.Pub specified in the manufacturer certificate. Further, the smart contract verifies that the OKP.Address is equal to the Tx.sender address, and, if everything is correct, SCA will store the OKP.Address linked to the SeK.Address defined in the certificate as trusted addresses. This way, there is no need to perform this process any more when identifying the SS in the future. It is also not needed to show the ownership of the SeK.Priv because the manufacturer certificate is proof enough that the owner of OK.priv is the only owner of SeK.priv. The pseudocode of the function *identification()* is shown in Algorithm 2.
**Algorithm 2** SCA identificationInternalInputsDevModelExternalInputsTx.sender,Cert     **if** !(Tx.sender⊂PA)     **return** No valid     $Cert_{raw},Signat\leftarrow Cert$     $Address_{Signer}\leftarrow recoverAddress\left(Cert_{raw},Signat\right)$     ManKP.Address←getManKP.Address()     **if** $\left(Address_{Signer}!=ManKP.Address\right)$     **return** No valid     **if** (Cert.DevModel!=DevModel)     **return** No valid    OKP.Address←getAddress(Cert.OKP.pub)     **if** (Tx.sender!=OKP.Address)     **return** No valid    SeKP.Address←getAddress(Cert.SeKP.pub)    addCryptodata(OKP.Address,SeKP.Address)**return**

#### 5.5.4. Sending Qualification Data (*SS → QualificationSC*)

When a SS’ certificate is validated, the SS can start transferring qualification data. Firstly, it will read the last block hash at slot *n* and will provide it to the Hardware Security Module (HSM) as a nonce. The HSM gathers real data from the sensor and signs it using the SeKP.priv together with the nonce. Then, the HSM sends the result to the Oracle controller. The last one generates a transaction to trigger the SCA’s function *receiveData()*, which is described in Algorithm 3 with the following inputs: real data, the signature, the number $N_{n}$, and the timestamp of the parent block of $N_{n}$, $\tau_{n-\theta }$. Then, the SS signs the transaction with OKP.priv and sends it to QualificationSC.
**Algorithm 3** QualificationSC receiveData$\mathrm{Internal}\mathrm{Inputs}T_{AA}$$\mathrm{External}\mathrm{Inputs}data,Signat,N_{n},\tau_{n-\theta }$     SeKP.Address←getCryptoData(Tx.sender)     **if** !(SeKP.Address)     **return** No valid     $nonce\leftarrow blockhash(N_{n})$     Content←(data||nonce)     $Address_{signer}\leftarrow recoverAddress\left(Content,Signat\right)$     **if** $\left(Address_{signer}!=SeKP.Address\right)$     **return** No valid     $\tau_{n+\beta }\leftarrow currenttime$     $\Delta \tau_{i}=\tau_{n+\beta }-\left(\tau_{n-\theta }-\Delta \tau_{AA}\right)$     $processData(Data,\Delta \tau_{i})$**return**

QualificationSC receives the transaction and authenticates it by checking the sender address (OKP.Address) in the SCA. If the SS is a trusted device, QualificationSC will receive a SeKP.Address from SCA. Then, from $N_{n}$, the smart contract obtains the block hash used as a nonce and verifies the SeKP signature. Finally, through using the time $\tau_{n-\theta }$, the time of the current block $\tau_{n+\beta }$ and $\Delta \tau_{AA}$ are defined in the setup phase, and QualificationSC can calculate the uncertainty interval $\Delta \tau_{i}$ (4), as well as estimate, when the measurement is gathered with a high reliability.

If the result is successful, the data would have proved to have DoA, DOI, DOTu, and DOF, and QualificationSC can process the data with all of the guarantees. Finally, the package receiver can read the qualification data and track it back to the data generator, thus obtaining DOTa.

## 6. System Implementation

**Figure 8:**
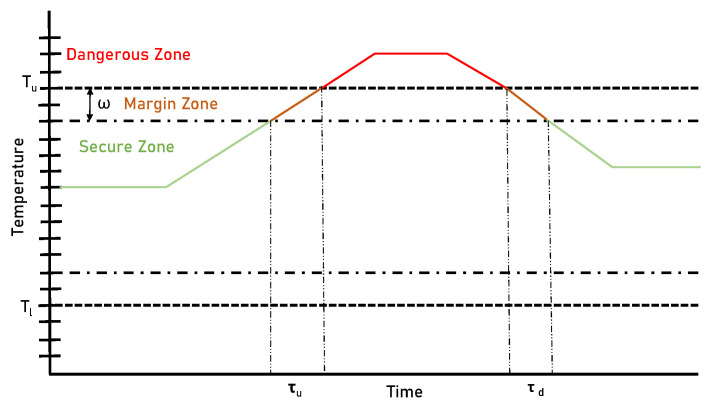
Graphical representation of the “Dangerous Zone”, “Margin Zone”, and “Secure Zone” of the temperature of a product in a cold chain.

In this section, we implement the infrastructure in a real use case. A packet in a cold chain must maintain a temperature between $T_{h}$ and $T_{l}$ within a safety margin ω. Additionally, we identified $\tau_{u}$ and $\tau_{d}$ as the times required to climb from the “Secure Zone” to the “Dangerous Zone” and vice versa, respectively, as seen in Figure 8. To ensure that the packet never enters the “Dangerous Zone”, we must take samples with a period less than $\tau_{p}$.

(6) $$\tau_{p}=\tau_{u}+\tau_{d}$$

From Mohamed Ahmed in [7], we take $T_{l}=+2$ °C and $T_{h}=+8$ °C, and we consider a safety margin of ω=1 °C. From [60,61], we set a continuous temperature change velocity ($V_{T}$) of a non refrigerated package of 0.1 °C/min.

With these data, we calculated $\tau_{p}$ = 20 min = 1200 s; therefore, we set 1200 s as the measurement period and 3 °C and 7 °C as the temperature limits in the qualification data. We considered 48 s as our assumable time $\Delta \tau_{AA}$ (i.e., γ=4). Also, we set 120 s as the maximum time for our transaction to be accepted (β = 10) [62], and we set θ as equal to 1 because skipped slots are very unlikely [58]. From (4), we obtained a minimum $\Delta \tau_{i}$ of 72 s with β=1, as well as a maximum of 180 s with β=10, which is much lower than the measurement period of 1200 s.

In the implementation, we used a real HSM, the same that was used by Dominic et al. in [24], the Blockchain Security 2Go starter kit R2 [63]. Moreover, it was connected to a low-price system on chip, Raspberry Pi 4B: Broadcom BCM2711, Quad core Cortex-A72 (ARM v8) 64-bit SoC @ 1.5 GHz 8 GB LPDDR4-3200 SDRAM. Through using a non optimized code, we obtained the results shown in Table 1, where *Get block hash* is the time needed to ask for the block hash of the latest block, *Data generation* represents the hash of the data and the signing operation by the SS, and *Tx generation* is the time taken to build the raw transaction and signing it for second time. Finally, the verification of the incoming data in QualificationSC has a cost of 21,830 gas, which, as of 22 July 2023, is equivalent to USD 0.86.

## 7. Security Analysis

In this section, we provide an analysis following the indications of the security requirements presented by Dan Liu et al. in [26] and explained in Section 2.1.

- DOAu: Every IoT node has a unique, irreplaceable, and irreplicable private key, which provides the IoT node with a unique address. Before accepting any data, the smart contract confirms that the sender address belongs to an accepted IoT node with a valid HSM (owner, manufacturer, and type).
- DOTa: Blockchains store all transaction histories with their sender address. Any entity with access to the blockchain can track the data back to the origin.
- DOI: Thanks to the use of a IoT device with hardware-based security, the SS and the measurements gathered from the environment are signed in the HSM even before they can be accessed by the controller of the IoT device. Then, these data and their signatures are verified by a smart contract thanks to our blockchain-based PKI. In this way, we achieve an end-to-end integrity protection of the data.
- DOTu: Once the identity is confirmed, QualificationCA receives the validated SeKP from SCA, and the smart contract verifies that the signature on the data was generated by SeKP before accepting the data, thus ensuring that the generator was a HSM in a SS. As indicated in Section 3.1, knowing the HSM in a SS that generates the data guarantees its trustworthiness.
- DOF: Guaranteeing data freshness is essential to avoid delay attacks and replay attacks. In delay attacks, the attacker generates a collection of correct measurements at time τ and uses them as measurements of other posterior times. Through using block hashes as nonces in the signatures, we can estimate a time slot where the data was generated over 72 s minimum and 180 s maximum with a probability of 99.98% in Ethereum (when considering an attacker investing USD six billion). The error margin can be divided by six by extending the assumable time $\tau_{AA}$ by 12 s.

## 8. Comparisons

In Table 2, we compare our work to the other solutions proposing Oracles to securely send data to blockchains. Notice that those works that do not consider the security of data origin, like [6,7,21,35,36], and they are no included in the comparison. As can be observed, Town Crier [13] and Astrea [14] have good security properties that make them good options for feeding smart contracts with trusted data, but they are not applicable to the IoT. The solution proposed by Jonathan [23] also has good security properties, and it does apply to the IoT; however, they were not capable of providing trustworthiness guarantees for the sensors gathering the data or the freshness of the data. Additionally, their solution requires a high-cost effort to enroll each IoT node. Our solution is the only one achieving this level of information security. Neither DiOr-SGX [22] or Alia Al S. et al. [20] provided mechanisms for blockchains to authenticate the IoT by sending the data, nor did they validate the IoT trustworthiness and the data freshness. Our proposed solution, in contrast to those previously discussed, not only successfully addresses all the security requirements, but it is also scalable and can be applied to IoT devices.

## 9. Conclusions

This paper presents a set of Ethereum smart contracts that performs the authentication and attestation of IoT devices and recognizes the timestamps of data collection. Usually, any IoT device’s owner controls the data collected. However, there are several use cases where a blockchain depends on sensor measurements, like blockchain-based supply chains, thus meaning the sensor owner could mislead the involved smart contracts. In our solution, the IoT device owner does not have any control over the IoT data. To achieve this, we developed an infrastructure where smart contracts receive measurements directly from sensors, the senders are authenticated, the hardware-based secure sensors are attested, and the data freshness is calculated before it is accepted for a low gas cost. In order to accomplish this, we measured the temperature using a hardware-protected IoT device, as well as designed a novel PKI to quickly authenticate IoT devices and their hardware-protected data on public blockchains without certificates. Moreover, we developed and analyzed the tools to demonstrate the freshness of the IoT data. In this research, we proved that it is possible to send non-manipulable data from IoT devices to smart contracts, and this is non-manipulable data even when controlling the IoT device. Thus, it paves the way for the creation of several new apps based on smart contracts, and it allows for the use of Ethereum in a variety of new scenarios involving IoT.

Still, the operation of our solution is closely dependent on the Ethereum blockchain, the mechanism of its operations, and it requires the use of a novel IoT sensor with a particular hardware architecture. Further investigation is needed to apply it to other blockchains like Hyperledger Fabric (where confidentiality can be added), or to Arbitrum (to achieve a better response time). On the other hand, the required IoT device implies more research in developing Secure Sensors before our solution can be applied to multiple and varied scenarios. Finally, our solution limits the sending data to raw measurement data. In the future, IoT remote attestation could be investigated to allow for some basic data preprocessing before uploading it to a blockchain.

## Figures and Tables

**Table 1:** Measurements of a non-optimized SS.

Operation	Time
*Get block hash*	140 ms
*Data generation*	303 ms
*Tx generation*	638 ms

**Table 2:** Comparison of Oracle protocols.

Oracle Protocols	Hardware Oracle Requirements						
	DOAu	DOTa	DOI	DOTu	DOF	Scalable	IoT Applicable
Town Crier [13]	✓	✓	✓	✓	✓	✓	✗
Astraea [14]	✓	✗	✓	✓	✓	✓	✗
DiOr-SGX [20]	✗	✗	✗	✗	✗	✓	✓
Jonathan [23]	✓	✓	✓	✗	✗	✗	✓
Alia [22]	✗	✓	✓	✗	✗	✓	✓
Our solution	✓	✓	✓	✓	✓	✓	✓

## Data Availability

Data are contained within the article.

## Abbreviations

The following abbreviations are used in this manuscript:

IoT	Internet of Things
HSM	Hardware Security Module
PKI	Public Key Infrastructure
DOAu	Data Origin Authenticity
DOTa	Data Origin Traceability
DOI	Data Origin Integrity
DOTu	Data Origin Trustworthiness
DOF	Data Origin Freshness
TLS	Transport Layer Security
CA	Certification Authority
SCA	Smart Certificate Authority
SS	Secure Sensor
API	Application Programming Interfaces
ECDSA	Elliptic Curve Digital Signature Algorithm
Priv	Private Key
Pub	Public Key
PoW	Proof of Work
PoS	Proof of Stake
UML	Unified Modeling Language
QualificationSC	Qualification Smart Contract
KP	Ke Pair
SeKP	Secure Element KeyPair
OKP	Owned KeyPair
ManKP	Manufacturer KeyPair
ManSC	Manufacturer Smart Contract
$\tau_{l}$	Time left. When a nonce becomes publicly known
$\tau_{r}$	Time right. When data is made publicly known
$\Delta \tau_{i}$	Uncertainty interval of data *i* generation
$\Delta \tau_{b}$	Block time. Time interval between two slots in Ethereum
$Rm_{n}$	RANDAO mix, random variable included in the block Published at slot *n*
$\tau_{n}$	Timestamp of slot *n*
$N_{n}$	Number of the blocks published at slot *n*
θ	Difference of the slots between the block $N_{n}$ and
	its parent block $N_{n}-1$
β	Number of slots between the one containing $Rm_{n}$
	and the one containing the signed data
PTA	PrevTime Attack to get $Rm_{n}$ before it is revealed
$\Delta \tau_{A}$	Quantity of time an attacker get $Rm_{n}$ in advance
	as a result of PTA
ξ	Probability of accidentally empty slots
μ	Probability of an attacker to be chosen as block proposer
$\Delta \tau_{AA}$	Quantity of time the solution assumed
	as a result of a possible PTA
γ	Number of slots intervals for reaching $\Delta \tau_{AA}$
$\Delta \tau_{NA}$	Difference between $\Delta \tau_{AA}$ and a higher $\Delta \tau_{A}$
Cer	Manufacturer Certificate
Tx.sender	Sender of a transaction
$T_{h}$	Highest secure temperature limit of a package
$T_{l}$	Lowest secure temperature limit of a package
